# Demographic, Clinical and Laboratory Characteristics of Ocular Syphilis: 6-Years Case Series Study From an Eye Center in East-China

**DOI:** 10.3389/fimmu.2022.910337

**Published:** 2022-06-10

**Authors:** Chuan-bin Sun, Geng-hao Liu, Rong Wu, Zhe Liu

**Affiliations:** ^1^Eye Center, Second Affiliated Hospital of Zhejiang University School of Medicine, Hangzhou, China; ^2^Custom Service Center, Second Affiliated Hospital of Zhejiang University School of Medicine, Hangzhou, China; ^3^Department of Ophthalmology, Zhejiang Provincial People’s Hospital, People’s Hospital of Hangzhou Medical College, Hangzhou, China

**Keywords:** ocular syphilis, uveitis, optic neuritis, optic atrophy, optical coherence tomography

## Abstract

**Purpose:**

To report the demographic, clinical, and laboratory characteristics of ocular syphilis based on a 6-year case series study from an eye center in East-China.

**Methods:**

A total of 131 cases (191 eyes) of ocular syphilis and the annual number of total syphilis cases from January 2016 to December 2021, were included in this study. Detailed medical records including systemic and ophthalmic medical history, a complete ophthalmic examination, color fundus photography, B-type ultrasound, fundus fluorescein angiography (FFA), spectral domain optical coherence tomography (SD-OCT), laboratory tests of the serum and cerebrospinal fluid (CSF) samples, as well as visual field test and orbital or cranial MRI in cases with suspected optic neuritis or optic atrophy, were collected and analyzed. Pearson Chi-square or Fisher’s exact tests was used for statistics analysis.

**Results:**

Of the 131 cases with ocular syphilis, 86 cases were in men and 6 cases had a past medical history or systemic manifestation of syphilis. HIV was found in only 2 of 70 cases undergoing serum HIV test. The average age was 54.0 years, ranging from 26–85 years. The average percentage of ocular syphilis out from the total syphilis cases was 5.1%, the average titer of serum rapid plasma regain (RPR) at presentation was 1:32, ranging from 1:1–1:512. The most predominant manifestation of ocular syphilis was posterior uveitis, followed by optic neuritis, optic atrophy, panuveitis, retinal vasculitis, and retinitis. The median of BCVA of all 191 eyes was 20/200 (ranging from no light perception to 20/20), and 20/40 (ranging from no light perception to 20/20) at presentation and final follow-up, respectively. Ocular syphilis with active inflammation responded well to penicillin therapy, no matter the initial visual acuity, ocular disease type, or RPR titers, as long as it was diagnosed early and treated properly and promptly. However, cases with optic atrophy, acute retinal necrosis, late diagnosis, permanent disruption, or loss of outer segment of photoreceptors of macular retina on SD-OCT showed poor visual improvement after therapy.

**Conclusions:**

Early diagnosis of ocular syphilis is still challenging in clinical practice and syphilis tests should be routinely performed in patients with uveitis, retinitis, optic neuritis, and optic atrophy.

## Introduction

Syphilis is a sexually transmitted disease caused by treponema pallidum infection. Although there was a dramatic decrease in syphilis cases due to the wide utility of the efficient penicillin G therapy during the 20th century, a resurgence of syphilis has been reported both in developed and developing countries since early 2000s (1–3). Ocular involvement is rare in syphilis infection. It is reported that ocular involvement could occur at any of three stages—primary, secondary, and tertiary—of syphilis, despite predominantly occurring in secondary and tertiary syphilis and their latent phase (1–3).

The visual prognosis of ocular syphilis is usually good if it is diagnosed early and treated properly and promptly. Pratas et al. reported that 75% of ocular syphilis cases had a good final visual outcome of best-corrected visual acuity (BCVA) ≥20/40 (1). Unfortunately, ocular syphilis may occasionally present as the only clinical manifestation of syphilis, and mimic other ophthalmic diseases such as non- infectious uveitis or optic neuritis, and therefore, can be easily misdiagnosed or erroneously treated with steroids which probably leads to irreversible visual loss (4, 5). Gu et al. reported that 23.5% of the late syphilis cases they researched met the WHO definition of blindness prior to treatment and patients with optic atrophy before treatment showed permanent visual loss. Hence, early diagnosis of ocular syphilis is vital to avoid irreversible visual loss (3).

In this study, we reviewed and evaluated the clinical data of 131 cases of ocular syphilis diagnosed in our eye center during a six year period and summarized its demographic, clinical, and laboratory characteristics, which may facilitate the early diagnosis of ocular syphilis and help to further understand its natural course and prognosis.

## Methods

### Subjects

In this study, 131 cases of ocular syphilis diagnosed in Eye Center, Second Affiliated Hospital of Zhejiang University School of Medicine from January 2016 to December 2021 were included in this study. The diagnosis of ocular syphilis was based on positive serum test results both of treponema pallidum particle agglutination (TPPA), rapid plasma regain (RPR), or toluidine red unheated serum test (TRUST), as well as ophthalmic examinations. Exclusion of congenital syphilis was performed before each case was included. The annual number of total syphilis cases diagnosed in the Second Affiliated Hospital of Zhejiang University School of Medicine between January 2016 and December 2021 were also collected to evaluate the percentage of ocular syphilis from the total syphilis cases at each year.

Detailed medical records including systemic and ophthalmic medical history, complete ophthalmic examination, color fundus photography, B-type ultrasound, fundus fluorescein angiography (FFA), spectral domain optical coherence tomography (SD-OCT), laboratory tests of the serum, and cerebrospinal fluid (CSF) samples, as well as visual field test and orbital or cranial MRI in cases with suspected optic neuritis or optic atrophy, were collected and analyzed. This study was conducted according to the tenets of the Declaration of Helsinki. Informed consents were obtained from all patients. Institutional review board approvals were obtained from Second Affiliated Hospital of Zhejiang University School of Medicine.

### Ophthalmic Examination

All 131 patients underwent BCVA, complete ophthalmic examination including slit-lamp examination and ophthalmoscopy, color fundus photography, visual field test, FFA, and SD-OCT. Color fundus photography was taken using Canon CX-1 (Canon Company, Japan), visual field was tested using 30 programs for Octopus 900 perimeter (HAAG-STREIT Diagnostics, Swissland), low vision program was used for patients with BCVA lower than 20/200, but better than hand motion. FFA was performed according to routine procedures using Spectralis HRA (Heidelberg Engineering GmbH, Germany). SD-OCT was performed using with radial linear scan (10 mm in length) centered at fovea for outer retina analysis by RTVue XR (Optovue Inc, USA).

### Orbital or Cranial MRI Examination

Patients with suspected optic neuritis, optic atrophy, or cranial nerve III, VI paralysis underwent orbital or cranial MRI examination using T1, T2-weighted imaging sequence with fat suppression and T1-weighted imaging sequence with gadolinium-enhancement.

### Serum and CSF Test

All 131 patients underwent serum tests including TPPA and RPR (or TRUST). Seventy cases also underwent serum tests for IgM or IgG antibodies of other pathogens including herpes viruses, hepatitis viruses, toxoplasma gondii, and HIV. Forty-eight patients underwent lumbar puncture for CSF tests including TPPA, RPR (or TRUST), white cell counting, and protein quantity. All patients underwent at least another serum test with an interval of 3 months after the initial antibiotic therapy.

### Antibiotic Therapy

All 131 patients (191 eyes) were referred to dermatologists for antibiotic therapy and 63 cases underwent standard neurosyphilis treatment regimen, i.e., intravenous injection of penicillin G 4~6 million units per 6 hours for 2 weeks, followed by intramuscular injection of penicillin G benzathine 24 million units per week for 3 weeks. 62 cases underwent intramuscular injection of penicillin G benzathine 2.4 million units per week for 3 weeks. The other 6 cases were treated with intravenous injection of ceftriaxone 2.0 per 12 hours for 2 weeks, among which, three cases showed positive skin test results for penicillin.

Topical steroid with or without cycloplegic eyedrops were routinely used in patients with active uveitis or stromal keratitis. Oral steroid or peribulbar injection of triamcinolone was used in 22 cases before the ocular syphilis was diagnosed but not continued after the diagnosis was confirmed. However, short-term oral steroid therapy (usually 3 to 5 days) was used in some cases by dermatologists to avoid Koch reaction.

During follow-up, 12 cases with initial penicillin G benzathine therapy and 2 cases with initial ceftriaxone therapy underwent deterioration or recurrence of ocular syphilis and were then treated with another course of therapy or transferred to intravenous penicillin G therapy. Daunomycin was used in one case to substitute penicillin G benzathine due to the reoccurrence of ocular syphilis and his refusal to another intramuscular injection of penicillin G benzathine, not allergic reaction.

### Grouping and Statistics Analysis

To evaluate the influence of ocular inflammation activity on the prognosis of ocular syphilis, 191 eyes with ocular syphilis were divided into active ocular inflammation group (125 eyes) and optic atrophy group (66 eyes). To evaluate the influence of diagnosis delay (the interval between ocular symptom occurrence and the diagnosis of confirmed ocular syphilis) on the prognosis of ocular syphilis, 125 eyes in the active ocular inflammation group were further divided into early (diagnosis delay ≤1 month, 75 eyes), moderately-delayed (diagnosis delay ≤3 months, 34 eyes), and late (diagnosis delay>3 months, 16 eyes) diagnosis groups. BCVA of eyes with ocular syphilis was divided into four groups: Group1: BCVA≥ no light perception, <20/200; Group2: BCVA≥20/200, <20/67; Group3: BCVA≥20/67, <20/40; Group4: BCVA≥20/40, ≤20/20. The difference between groups was compared using Pearson Chi-square or Fisher’s exact tests with *p* values of <0.05 considered to be statistically significant.

## Results

### Domestic Characteristics of Patients With Ocular Syphilis

A total of 2,562 syphilis cases were confirmed between January 2016 and December 2021 in this study, of which 131 patients (191 eyes) were diagnosed as ocular syphilis. Of 131 cases with ocular syphilis, only 6 cases had a medical history or clinical manifestations of systemic syphilis, including dermal rashes in 4 cases, syphilic epilepsy, and dementia in one case, respectively. No past medical history of, or systemic manifestations indicating syphilis were reported in the other 125 patients. Of 70 cases undergoing serum tests for antibodies of other pathogens including HIV it was detected only in 2 cases with ocular syphilis.

**Figure 1:**
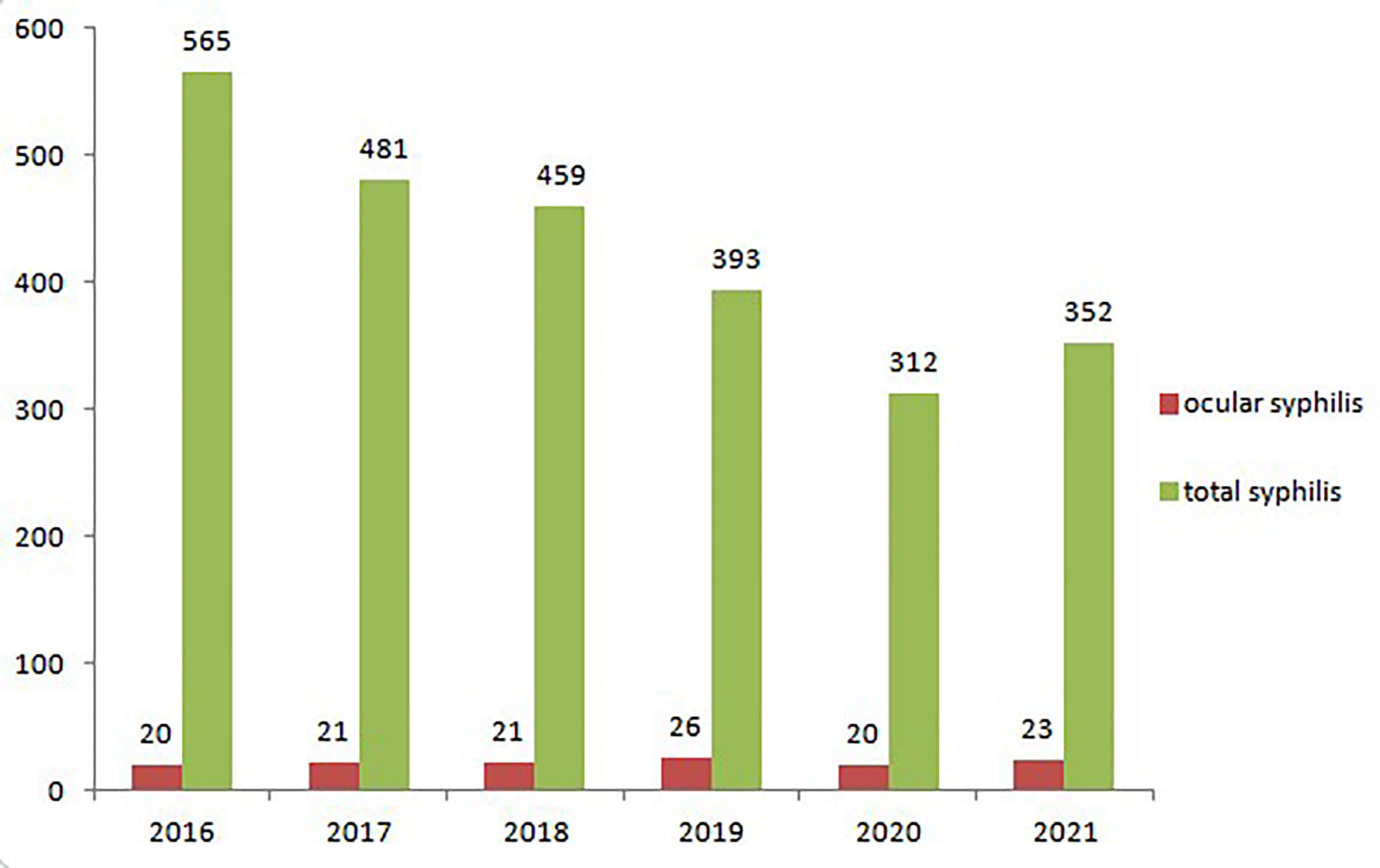
Annual numbers of ocular syphilis and total syphilis cases.

The annual number of ocular syphilis and total syphilis cases were 20 and 565 in 2016, 21 and 481 in 2017, 21 and 459 in 2018, 26 and 393 in 2019, 20 and 312 in 2020, and 23 and 352 in 2021, respectively (**Figure 1**). Th e average percentage of ocular syphilis out of total syphilis cases was 5.1%, with an annual distribution of 3.5% in 2016, 4.4% in 2017, 4.6% in 2018, 6.6% in 2019, 6.4% in 2020, and 6.5% in 2021. Of 131 cases with ocular syphilis, 86 cases were men and 45 cases were women. The average age was 54.0 years (ranging from 26 to 85 years).

### Ophthalmic Characteristics of Eyes With Ocular Syphilis

The ophthalmic manifestations of 131 patients (191 eyes) with ocular syphilis are shown in **Table 1**. The most predominant manifestation of ocular syphilis was posterior uveitis (54 cases, 67 eyes), followed by optic neuritis (48 cases, 60 eyes), optic atrophy (39 cases, 66 eyes), severe vitritis (22 cases, 30 eyes), panuveitis (15 cases, 22 eyes), retinal vasculitis (12 cases, 16 eyes), and retinitis (8 cases, 10 eyes). This was followed by cystoid macular edema (6 cases, 8 eyes), intermediate uveitis (6 cases, 7 eyes), anterior uveitis (5 cases, 5 eyes), papilloedema-like optic neuropathy (4 cases, 5 eyes), oculomotor paralysis (4 cases, 4 eyes), macular premembrane (3 cases, 4 eyes), keratitis (3 cases, 3 eyes), Argyll-Robertson pupil (2 cases, 4 eyes), secondary glaucoma (2 cases, 2 eyes), ischemic anterior optic neuropathy (1 case, 1 eye), inflammatory orbital pseudotumor (1 case, 1 eye), and abducens nerve paralysis (1 case, 1 eye).

**Table 1:** Clinical manifestations of ocular syphilis.

ocular manifestations	cases (eyes)
keratitis	3 (3)
anterior uveitis	5 (5)
granulomatous	3 (3)
non-granulomatous	2 (2)
severe vitritis	22 (30)
intermediate uveitis	6 (7)
posterior uveitis	54 (67)
posterior placoid chorioretinitis	39 (51)
AZOOR-like retinitis	11 (12)
panuveitis	15 (22)
retinitis	8 (10)
miliary retinitis	4 (5)
acute retinal necrosis-like retinitis	3 (4)
retinal vasculitis	12 (16)
retinal artery occlusion	1 (1)
retinal vein occlusion	4 (4)
optic neuritis	48 (60)
papillitis	40 (48)
retrobulbar optic neuritis	8 (12)
papilloedema-like optic neuropathy	4 (5)
ischemic anterior optic neuropathy	1 (1)
optic atrophy	39 (70)
oculomotor paralysis	4 (4)
abducens nerve paralysis	1 (1)
inflammatory orbital pseudotumor	1 (1)
cystoid macular edema	6 (8)
macular premembrane	3 (4)
Argyll-Robertson pupil	2 (4)
secondary glaucoma	2 (2)
Total	131 (189)

**Figure 2:**
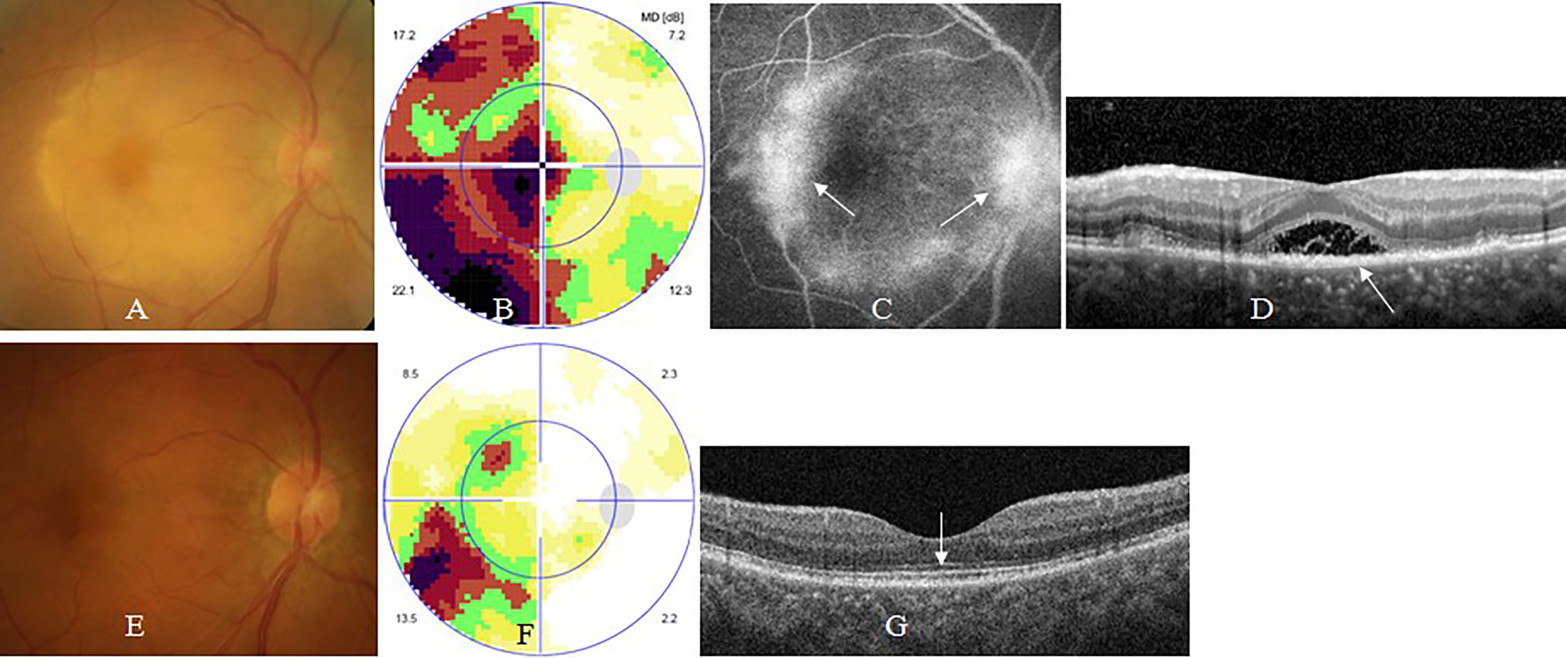
Syphilic placoid chorioretinitis. A 57-year old man complained of sudden visual loss in the right eye for 1 week. At presentation, his right eye had visual acuity of counting fingers, clear anterior segment and placoid edema of posterior retina **(A)**, large central scotoma on visual field test **(B)**, late-staged hyperfluorescence of posterior retinal and optic disc on fluorescein angiography (FFA) **(**arrow, **C)**, disruption or loss of outer segments of macular retina (arrow) and serous macular detachment on optical coherence tomography (OCT) **(D)**. Two months after antibiotic therapy, his visual acuity recovered to 20/25 in the right eye, with normal posterior retina **(E)**, small arcuate scotoma on visual field test **(F)**, and normal contour of outer segments of macular retina on OCT **(**arrow, **G)**.

**Figure 3:**
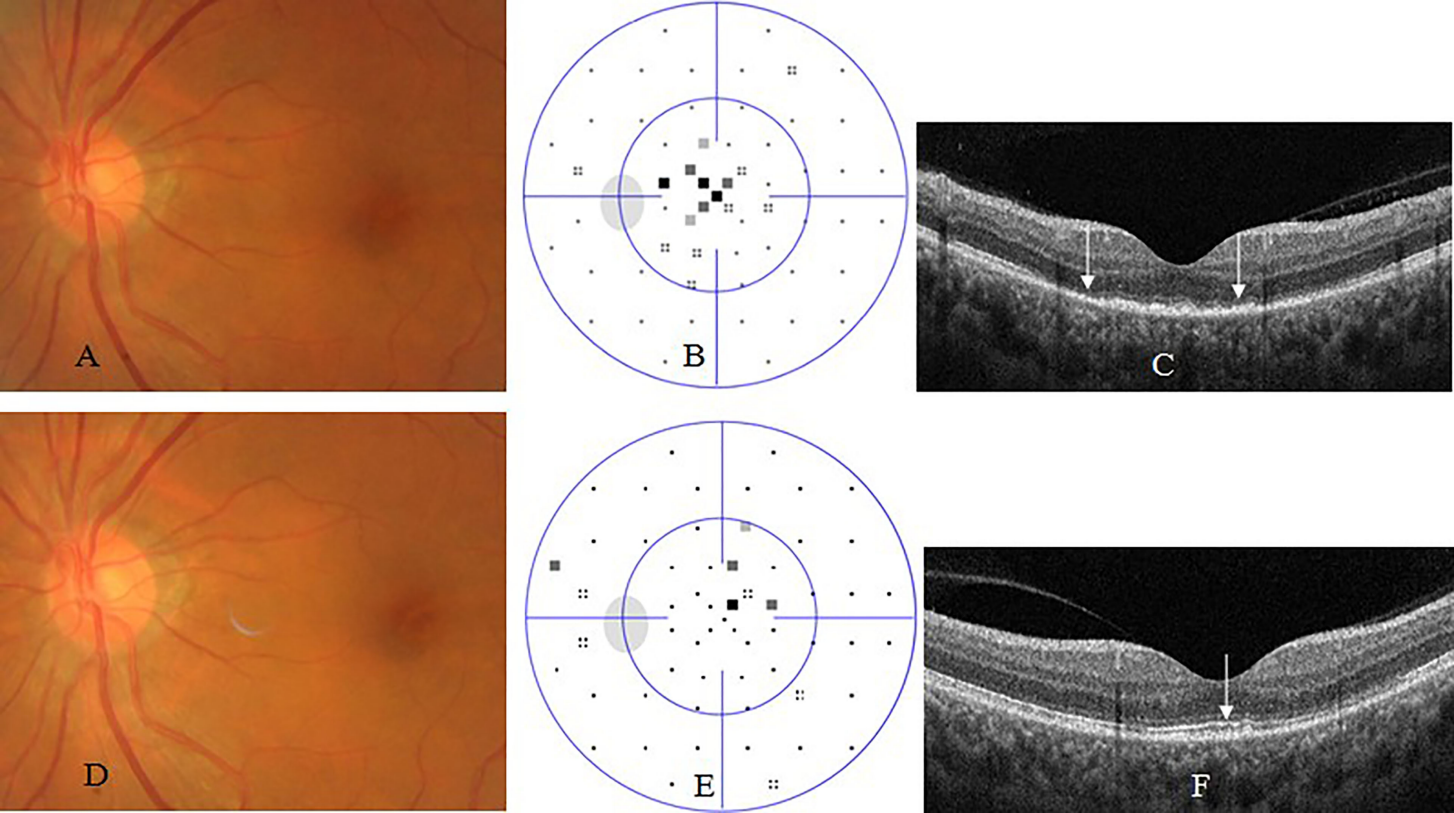
Acute occult outer retinopathy -like syphilic retinitis. A 54-year old woman complained of blurred vision in the left eye for 2 weeks. At presentation, his left eye had visual acuity of 20/40, clear anterior segment and normal fundus **(A)**, small central scotoma on visual field test **(B)**, and disruption or loss of outer segments of macular retina **(**arrow, **C)** on OCT. Two months after antibiotic therapy, her visual acuity recovered to 20/20 in the left eye, with normal fundus **(D)**, nearly normal visual field test **(E)**, and nearly complete recovery of outer segments of macular retina **(**arrow, **F)**.

Placoid chorioretinitis (39 cases, 51 eyes) was the most common subtype of posterior uveitis, followed by acute occult outer retinopathy (AZOOR)-like retinitis (11 cases, 12 eyes). Placoid chorioretinitis typically appeared as posterior yellowish retinal lesions with or without serous retinal detachment (**Figure 2**). AZOOR-like retinitis generally presented as normal fundus appearance under ophthalmoscopy (**Figure 3**). Uvea involvement also appeared as panuveitis, intermediate uveitis, and anterior uveitis.

**Figure 4:**
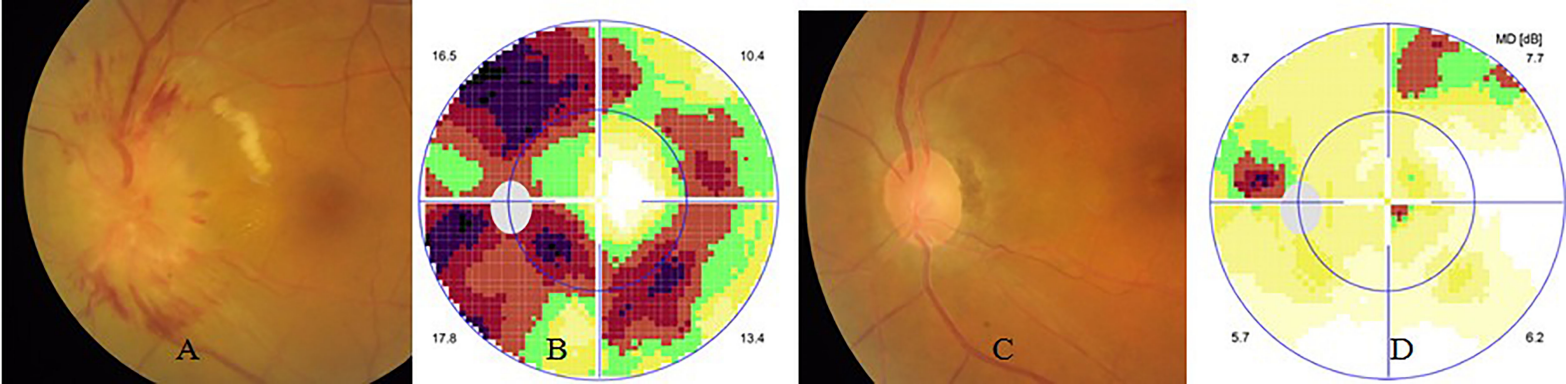
Syphilic optic neuritis. The left eye of the same case in **Figure 2**. At presentation, his left eye had visual acuity of 20/20, clear anterior segment and swollen optic disc **(A)**, and circular scotoma on visual field test **(B)**.Two months after antibiotic therapy, his visual acuity was still 20/20, with normal optic disc **(C)**, and nearly normal visual field test **(D)**.

Optic neuritis predominantly manifested as optic papillitis (40 cases, 48 eyes) (**Figure 4**) and occasionally as retrobulbar optic neuritis (8 cases, 12 eyes). Other rare optic neuropathies due to syphilis infection included papilloedema-like optic neuropathy and ischemic anterior optic neuropathy. Papilloedema-like optic neuropathy appeared as swollen optic disc, normal BCVA, and visual field but normal intracranial pressure, which can mimick optic disc vasculitis type I on ophthalmic manifestation.

Retina involvement included retinitis and retinal vasculitis and retinitis mainly appeared as miliary retinitis and acute retinal necrosis (ARN). Miliary retinitis manifested yellowish spots above retinal surface which might disappear after proper and prompt anti-syphilis therapy. Syphilic ARN mainly appeared as focal or diffuse peripheral yellowish necrotic lesions, accompanied by retinal vessels especially retinal artery narrowing, sheath on fundus ophthalmoscopy, and occlusion on FFA. Miliary retinitis occurred isolatedly or as part manifestation of syphilic ARN. Retinal vasculitis was sometimes accompanied by retinal artery or vein occlusion.

Coexistence of two or more ophthalmic diseases was common in ocular syphilis, most of which were found in optic papillitis cases accompanied by posterior placoid chorioretinitis, AZOOR-like retinitis, or retinal vasculitis. Vitritis was frequently accompanied by uveitis, retinitis, or even optic neuritis. Cystoid macular edema and macular premembrane were usually accompanied by uveitis, especially intermediate uveitis. Oculomotor paralysis and abducens nerve paralysis were accompanied with uveitis, optic nerve involvement, or inflammatory orbital pseudotumor.

As for cases with concurrent sysphilis and HIV, one case presented as severe vitritis, placoid chorioretinits, occlusive retinal vasculitis and pappilitis, the other case with concurrent syphilis and HIV infection presented as retinal vasculitis, which were similar to those with syphilis but without HIV infection.

### Ophthalmic Imaging and MRI Characteristics of Eyes With Ocular Syphilis

FFA showed early-staged hypofluorescence of and late-staged staining of retinal pigment epithelium cells in eyes with posterior placoid chorioretinitis or AZOOR-like retinitis. Fluorescein leakage from posterior or peripheral retinal vessels, and/or radial capillaries above optic disc were shown in eyes with retinal vasculitis, posterior placoid chorioretinitis, posterior or intermediate uveitis, and/or papillitis, respectively (**Figure 2**). Occlusion of retinal vessels and non-perfusion of retinal capillaries were shown in occlusive retinal vasculitis, especially in ARN.

SD-OCT revealed disruption or loss of the outer segment (i.e., ellipsoid zone and interdigitation zone) of photoreceptors of the retina, with or without triangle- or wedge- shaped precipitates above retinal pigment epithelium cells in eyes with placoid chorioretinitis or AZOOR-like retinitis at presentation, and complete or partial recovery of outer segment of photoreceptors of the retina accompanied with disappearance of precipitates after antibiotic therapy (**Figures 2**, **3**), thickening of and cystoid hyporeflection within macular retina, with or without foveal detachment in eyes with cystoid macular edema, as well as linear premacular hyperreflection in eyes with macular premembrane.

Visual field test showed central scotoma, arcuate, tubular visual defect, or generalized depression in eyes with optic neuritis or optic atrophy (**Figure 4**). AZOOR-like retinitis and placoid chorioretinitis suspected as retrobulbar optic neuritis usually demonstrated central scotomas (**Figures 2**, **3**).

Orbital or cranial MRI revealed optic nerve enlargement accompanied by optic nerve or sheath contrast enhancement in eyes with active-staged optic neuritis, whereas normal or thinned optic nerve was shown in eyes with early- or late- staged optic atrophy, respectively.

### BCVA of Eyes With Ocular Syphilis

BCVA of 131 patients (191 eyes) with ocular syphilis at presentation and final follow-up was shown in **Table 2**. The median of BCVA of all 191 eyes was 20/200 (ranged from no light perception to 20/20), and 20/40 (ranged from no light perception to 20/20) at presentation and final follow-up, respectively. Also, 22 cases misdiagnosed as non-infectious uveitis, retinitis, or optic neuritis were treated with oral steroid or peribulbar injection and then underwent visual deterioration. However, most cases achieved improvement in visual acuity and ocular conditions after antibiotic therapy, except for a few cases with severe ARN, optic atrophy, or retinal artery occlusion.

**Table 2:** Comparison of visual acuity between two groups of ocular syphilis eyes at presentation and final follow-up.

		active ocular inflammation group (eyes)		optic atrophy group (eyes)	
BCVA		presentation	final follow-up	presentation	final follow-up
Group 1	≥NLP, <20/200	46	16	40	34
Group 2	≥20/200, <20/67	41	10	10	11
Group 3	≥20/67, <20/40	14	12	6	6
Group 4	≥20/40, ≤20/20	24	87	10	15
Total		125	125	66	66

BCVA, best corrected visual acuity; NLP, no light perception.

By subgroup analysis, BCVA within Group 1, Group 2, Group 3, and Group 4 was 46, 41, 14, and 24 eyes; and 40, 10, 6, and 6 eyes at presentation, as well as 16, 10, 12, and 87 eyes; and 34, 11, 6, and 15 eyes at final follow-up, in active ocular inflammation group and optic atrophy group, respectively (**Table 2**). The difference was statistically significant (*p*<0.05), indicating ocular syphilis eyes with active ocular inflammation achieved more BCVA improvement than those with optic atrophy after antibiotic therapy.

Among ocular syphilis eyes with active ocular inflammation, BCVA within Group 1, Group 2, Group 3, and Group 4 was 22, 32, 6, and 15 eyes; 14, 9, 5, and 6 eyes; and 10, 0, 3, and 3 eyes at presentation, as well as 6, 3, 6, and 60 eyes; 5, 3, 4, and 22 eyes; and 5, 4, 2, and 5 eyes at final follow-up, in early, moderately-delayed, and late diagnosis group, respectively (**Table 3**). The difference was statistically significant (*p*<0.05), indicating the early and moderately-delayed diagnosis groups achieved more BCVA improvement than the late diagnosis group.

**Table 3:** Comparison of visual acuity among ocular syphilis eyes with different diagnosis delay.

		early diagnosis (eyes)		moderately-delayed diagnosis (eyes)		late diagnosis (eyes)	
BCVA		presentation	final follow-up	presentation	final follow-up	presentation	final follow-up
Group 1	≥NLP, <20/200	22	6	14	5	10	5
Group 2	≥20/200, <20/67	32	3	9	3	0	4
Group 3	≥20/67, <20/40	6	6	5	4	3	2
Group 4	≥20/40, ≤20/20	15	60	6	22	3	5
Total		75	75	34	34	16	16

BCVA, best corrected visual acuity; NLP, no light perception; early diagnosis, diagnosis delay ≤ 1month; moderately-delayed diagnosis, diagnosis delay> 1month, ≤ 3months; late diagnosis, diagnosis delay> 3months.

BCVA better than 20/40 was found in 69.6% (87/125) of eyes with active ocular inflammation, 22.7% (15/66) of eyes with optic atrophy, and 53.4% (102/191) of total eyes with ocular syphilis after antibiotic therapy. Whereas BCVA better than 20/67 was found in 79.2% (99/125) of eyes with active ocular inflammation, 31.8% (21/66) of eyes with optic atrophy, and 62.8% (120/191) of total eyes with ocular syphilis after anti-syphilis therapy. BCVA better than 20/40 was found in 80% (60/75) of eyes with early diagnosis, 64.7% (20/34) of eyes with moderately-delayed diagnosis, and 19.2% (5/16) of eyes with late diagnosis in active ocular inflammation group.

At final follow-up, 19 eyes in active ocular inflammation group showed BCVA ≤ 20/200. Among them, BCVA got worse in 7 eyes which included 4 eyes with ARN-like retinitis, 2 eyes with posterior placoid chorioretinitis, and 1 eye with cystoid macular edema secondary to intermediate uveitis. BCVA kept stable or improved slightly in the other 12 eyes which included 3 eyes with posterior placoid chorioretinitis, 2 eyes with cystoid macular edema secondary to intermediate uveitis, 2 eyes with late diagnosed optic neuritis, 2 eyes with combined ARN-like retinitis and posterior placoid chorioretinitis, 1 eye with AZOOR-like retinitis, 1 eye with branch retinal artery occlusion, and 1 eye with refractory glaucoma secondary to panuveitis. Permanent disruption or loss of outer segment of photoreceptors of macular retina on SD-OCT was found in eyes with posterior placoid chorioretinitis, AZOOR-like retinitis, ARN, or cystoid macular edema. Retinal vascular occlusion involving macular retina on FFA was found in eyes with ARN or branch retinal artery occlusion.

### Serum and CSF Tests

**Figure 5:**
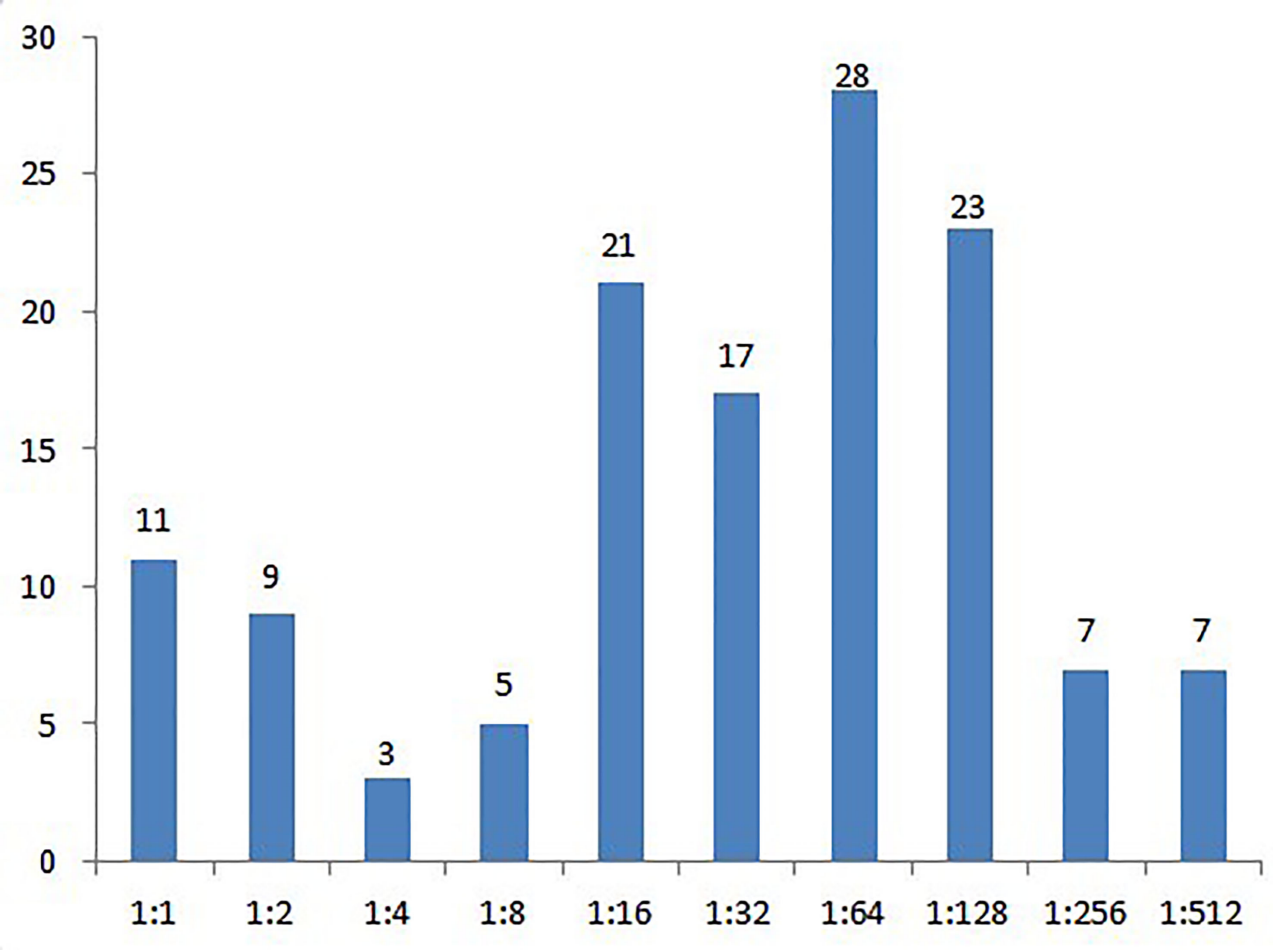
Rapid plasma regain titers of cases with ocular syphilis at presentation.

The average titer of serum RPR (or TRUST) among 131 cases at presentation was 1:32, ranging from 1:1 to 1:512 (**Figure 5**). At final follow-up, the titer of serum RPR dropped at least 4 fold or stayed lower than 1:2 and was in 120 out of 131 cases after antibiotic therapy. However, negative serum RPR (or TRUST) result was detected only in one case. Of the 70 cases that also underwent serum tests for antibodies of other pathogens, HIV IgG was detected in 2 cases which were subsequently confirmed with HIV infection of 48 cases that underwent lumbar puncture and CSF tests, positive TPPA and RPR (or TRUST) results were found in 32 cases, with a mean titer of RPR 1:4 (ranged from 1:1 to 1:160). CSF white blood cells ≥ 8 x 10^6^/L was found in 32 cases, CSF protein ≥ 0.45 g/L in 26 cases, and both were found in 19 cases.

## Discussion

Syphilis is generally recognized as a great imitator and could mimic nearly all systemic diseases. Hence, early diagnosis and prompt treatment were challenging in clinical practice. Compared to systemic syphilis, ocular involvement is rare. In this study, ocular syphilis occurs in only 5.1% of total syphilis cases based on a 6 year case series study, which is similar to previous studies reported in literature (1, 5, 6). Although ocular syphilis mainly occurs in secondary and tertiary syphilis, our study demonstrated that 95.4% of syphilis cases (125/131) had no past medical history, or systemic manifestation of syphilis at presentation, which indicates that most ocular syphilis may present as the only clinical manifestation of syphilis, i.e., most ocular syphilis may occur in latent syphilis (7, 8).

Different from ocular syphilis in developed western countries which frequently occur in HIV positive patients (ranged from 22% to 65%), ocular syphilis was mostly found in HIV negative patients in China and other East-Asian countries (5–12). In our study, only 2.9% (2/70) of ocular syphilis cases were HIV positive, the prevalence of concurrent ocular syphilis and HIV was similar to that reported in previous investigation from China, indicating that despite a high risk of co-infection in syphilis cases, concurrent HIV infection rate might be low in China (5).

Recently, syphilis resurged all over the world and ocular syphilis has received more attention because of its possibility to cause blindness. The first reason ocular syphilis is recognized as sight-threating is that it is easily misdiagnosed as a non-infectious eye disease and then erroneously treated with a systemic or local steroid which might cause irreversible visual loss. Deterioration of uveitis or optic neuritis and decreased vision were frequently reported in ocular syphilis patients misdiagnosed as non-infectious ocular inflammations (4, 5). In this study, 22 cases were erroneously treated with oral steroid or peribulbar injection before ocular syphilis was diagnosed and all underwent visual deterioration. However, most cases underwent improvement in visual acuity and ocular conditions after antibiotic therapy, except a few cases with severe ARN, optic atrophy, or retinal artery occlusion.

Additionally, late diagnosis was another major cause of permanent visual loss in eyes with ocular syphilis (3, 5). Gu et al. reported that visual improvement was achieved only in 35.8% (24/67) of eyes after antibiotic therapy and eyes with optic atrophy prior to treatment showed little visual improvement (3). Our results in this study reveal similar findings as 69.6% of eyes with active ocular inflammation, compared to 22.7% of eyes with optic atrophy at presentation, achieved BCVA better than 20/40 after treatment. Moreover, 80% of eyes with early diagnosis compared to19.2% of eyes with late diagnosis in active ocular inflammation group, achieved BCVA better than 20/40 after treatment. Hence, our findings further support that early diagnosis is vital to avoid permanent visual loss for ocular syphilis cases.

In this study, most ocular syphilis (103/131) occurred in patients with a RPR titer more than 1:16 at presentation, indicating ocular syphilis was apt to occur in cases with higher RPR titer. Gu et al. reported similar findings that ocular syphilis occurred more predominantly in patients with a RPR titer>8 (3). However, no relationship was found among RPR titers, ocular manifestations, therapeutic efficiency, and visual prognosis in our study.

Although lumbar puncture and CSF tests were not mandatory for the diagnosis of ocular syphilis and therapy regimen selection, positive TPPA and RPR results, as well as positive findings in white blood cells counting or (and) protein quantification in CSF tests facilitated understanding whether the brain was involved in choosing a standard neurosyphilis treatment regimen rather than only intramuscular penicillin G benzathine therapy. The latter was frequently used in ocular syphilis therapy, as reported in our study and by Kim and Lavery (12, 13). Unfortunately, some cases treated with only intramuscular penicillin G benzathine therapy underwent deterioration or recurrence of ocular syphilis and needed another course of therapy or should be transferred to standard intravenous penicillin G regimen as long as they were not allergic to penicillin G.

Ocular syphilis nearly involved every ophthalmic and orbital tissues, posterior or panuveitis, optic neuritis, optic atrophy, retinitis, and retinal vasculitis and were the most predominant manifestations of ocular syphilis reported in literature, similar to our findings in this study (3, 5, 7–10, 14). Hence, syphilis should be routinely excluded in cases with ocular or orbital inflammations, especially when steroid therapy did not ameliorate or even deteriorate the ophthalmic conditions.

Previous investigations revealed that ocular syphilis eyes with uveitis showed a good response to penicillin G treatment and better visual improvement than those with optic atrophy and that delay in treatment also indicated poor visual prognosis (3, 5, 14–16). In our study, except ARN, most ocular syphilis with active inflammation responded well to standard penicillin G therapy, no matter what the initial BCVA, ocular disease type, and RPR titers were as long as it was diagnosed early and treated properly. However, cases with optic atrophy, late diagnosis, ARN, permanent disruption or loss of outer segment of photoreceptors of macular retina on SD-OCT showed little visual improvement after therapy (3, 5, 17). Therefore, ARN, optic atrophy, late diagnosis, inadequate therapy, permanent disruption or loss of outer segment of photoreceptors of macular retina on SD-OCT, may indicate a poor visual prognosis in eyes with ocular syphilis. This finding may further facilitate our understanding about the visual prognosis of ocular syphilis.

In summary, considering that ocular syphilis may occur without a past medical history, or even systemic manifestation of syphilis, early diagnosis of ocular syphilis is still challenging in clinical practice. Hence, the syphilis test is highly recommended as a routine assay in patients with uveitis, retinitis, optic neuritis, and even optic atrophy.

## Data Availability Statement

The original contributions presented in the study are included in the article/supplementary material. Further inquiries can be directed to the corresponding author.

## Ethics Statement

The studies involving human participants were reviewed and approved by Second Affiliated Hospital of Zhejiang University School of Medicine. The patients/participants provided their written informed consent to participate in this study.

## Author Contributions

CBS and ZL wrote and reviewed the manuscript, GHL and RW collected and analyzed the patient data. All authors read and approved the final manuscript.

## Funding

This study was supported in part by Ophthalmology Star Program (QMX2019-01-001). The funding organization does not have any role in the design or conduct of this study.

## Conflict of Interest

The authors declare that the research was conducted in the absence of any commercial or financial relationships that could be construed as a potential conflict of interest.

## Publisher’s Note

All claims expressed in this article are solely those of the authors and do not necessarily represent those of their affiliated organizations, or those of the publisher, the editors and the reviewers. Any product that may be evaluated in this article, or claim that may be made by its manufacturer, is not guaranteed or endorsed by the publisher.
